# The Effects of Taping Combined with Wrist Stabilization Exercise on Pain, Disability, and Quality of Life in Postpartum Women with Wrist Pain: A Randomized Controlled Pilot Study

**DOI:** 10.3390/ijerph18073564

**Published:** 2021-03-30

**Authors:** Kyoung-Sim Jung, Jin-Hwa Jung, Hyung-Soo Shin, Jae-Young Park, Tae-Sung In, Hwi-Young Cho

**Affiliations:** 1 Department of Physical Therapy, Gimcheon University, Gimcheon 39528, Korea; 20190022@gimcheon.ac.kr; 2 Department of Occupational Therapy, Semyung University, Jecheon 27136, Korea; otsalt@semyung.ac.kr; 3 Department of Physical Therapy, Kyungwoon University, Gumi 39160, Korea; hsshin@ikw.ac.kr (H.-S.S.); hopi7@ikw.ac.kr (J.-Y.P.); 4 Department of Physical Therapy, College of Health Science, Gachon University, Incheon 21936, Korea

**Keywords:** postpartum, wrist pain, stabilization exercise, taping

## Abstract

The purpose of this study was to evaluate the effects of wrist stabilization exercise combined with taping on wrist pain, disability, and quality of life in postpartum women with wrist pain. Forty-five patients with wrist pain were recruited and randomly divided into three groups: wrist stabilization exercise + taping therapy (WSE + TT) group (n = 15), wrist stabilization exercise (WSE) group (n = 15), and control group (n = 15). The WSE + TT and WSE groups performed wrist stabilization exercises for 40 min (once a day, five times a week for eight weeks), and the control group performed passive range of motion (P-ROM) exercise for the same amount of time. Additionally, the WSE + TT group attached taping to the wrist and forearm during the training period. The visual analogue scale (VAS) was used to assess pain level of the wrist. The Disabilities of the Arm, Shoulder and Hand (DASH) and the Short Form-36 (SF-36) were used to evaluate the degree of wrist disability and quality of life, respectively. The WSE + TT group showed a significant decrease in wrist pain and functional disability compared to two groups (*p* < 0.05). Significant improvement in the SF-36 score was observed in the WSE + TT and WSE groups compared to that in the control group (*p* < 0.05). However, there was no significant difference between the WSE + TT and WSE groups in the SF-36. Our findings indicate that wrist stabilization exercise combined with taping is beneficial and effective in managing wrist pain and disability in postpartum women with wrist pain.

## 1. Introduction

Physical problems in women after childbirth are considered a risk factor for health and emotional well-being, and musculoskeletal pain in various areas, such as the back, neck, and shoulders, is common [1]. The wrist is a biaxial joint that is placed in various positions during functional activity [2] and is exposed to various types of stress such as load pressure, torsion, and traction [3]. Wrist pain that occurs after childbirth is called “de Quervain’s disease” or “baby wrist” and is common in clinical practice [4]. The laxity of the peripheral joints increases during pregnancy [5], which is known as a risk factor for joint pain and damage [6]. In addition, abuse of the wrist, such as lifting or carrying the baby repeatedly and for a long time and wrapping the baby while breastfeeding, causes muscle strain around the wrist joint [7].

In a study that investigated the pattern and cause of wrist pain in women after childbirth, 57.5% of women had wrist pain after childbirth, and 84% reported that it lasted more than two months after childbirth. In addition, the pain pattern was bilateral, most of the symptoms were expressed on the radial side of the wrist, and it was reported that the risk of wrist pain was more than twice as high in the case of breastfeeding [5]. These symptoms develop into chronic pain, which can cause serious dysfunction, quality of life problems, and reduced work time; therefore, appropriate management is necessary [8].

Taping intervention is easy to apply, can be applied to various parts of the body, and provides fixation and protection to the applied part. Thus, it has been used for the management of pain and swelling caused by acute injury and for the purpose of preventing damage to joints and soft tissues in the clinic and sports fields [9]. However, in the past 20 years, various application methods and physiological effects of taping have been reported in various types of patients and subjects. According to the previous studies, taping methods show positive effects on the alleviation of pain caused by muscle fatigue [10], the increase of gait and balance function in strokes [11,12], and the management of pain and functional disability in patients with epicondylitis [13], wrist pain [14], and hand osteoarthritis [15]. Interestingly, however, some studies reported that therapeutic exercise combined with taping improves pain relief and motor function more effectively than exercise only [11,13]. In addition, in a review study on the effect of upper extremity muscle strength training, it was found that pain was significantly improved compared to that in the control group, which performed only simple fixation of the wrist. In order to manage musculoskeletal disorders and joint damage, such as of the elbows, wrists, and hands, it is important to select an exercise type suitable for the joint condition [16].

Stabilization exercise provides stability to unstable joints by improving the balance of the muscles around the joint and muscle activation [17,18]. Although several studies reported that stabilization exercise effectively relieves pain and disability following musculoskeletal problems such as low back pain and osteoarthritis [19,20], there was no clinical trial to determine the effectiveness of stabilization exercise on wrist pain and disability. In addition, the effect of the combination of stabilizing exercise and taping on the management of various symptoms caused by the joint laxity due to childbirth is unclear.

Therefore, this study aimed to investigate the effects of wrist stabilization exercise with taping on wrist pain, disability, and quality of life in postpartum women with wrist pain.

## 2. Materials and Methods

### 2.1. Participants

Forty-five postpartum women with wrist pain living in Gimcheon City, South Korea participated in this study. All subjects included in the study had a visual analogue scale (VAS) of 30 mm or higher, had never experienced wrist pain before pregnancy, and had given birth within 1 year. Subjects taking pain-related medications or receiving other treatments were excluded. Informed consent was voluntarily obtained from all subjects before participation in our study, which was approved by the Institutional Review Board (IRB) of Gachon University (IRB no. 1044396-202010-HR-184-01) and enrolled in the clinical research information services that comply with the World Health Organization international clinical trial registry platform (clinical trial number: KCT0006011). We used G*power 3.1.9.4 software (Heinrich-Heine-University Düsseldorf, version 3.1.9.4, Düsseldorf, Germany) to calculate the sample size. In the present study, the mean power was set at 0.8 and the alpha error at 0.05. The effect size was set to 0.7168604 based on the pilot study (15 subjects). The analysis of G*power software shows that at least 36 participants should make an acceptable group sample size; thus, 45 participants were recruited in consideration of drop-out.

### 2.2. Protocol

**Figure 1:**
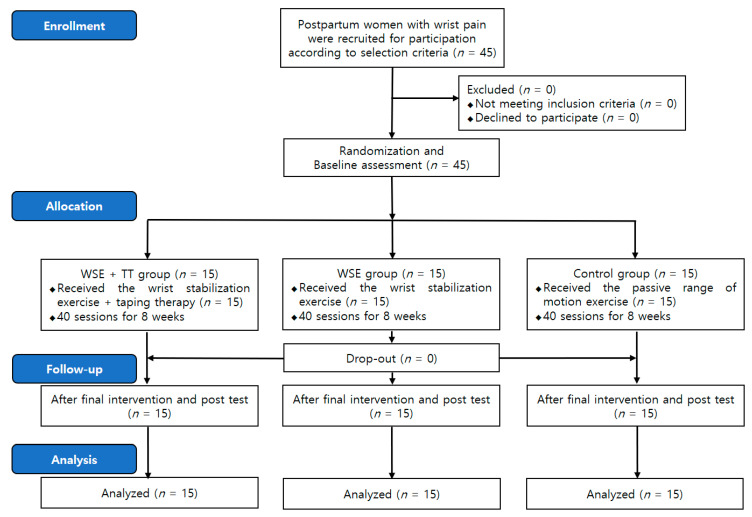
Flow diagram of participants throughout the study.

This study was designed as a double-blinded and randomized controlled trial (RCT) pilot study. To recruit participants, a poster was attached to various community organizations such as the obstetrics and gynecology clinic, women’s welfare centers, postpartum care centers, apartments, and the community-service center located in Gimcheon city. In addition, guidance documents for the recruitment of research subjects were promoted to the local online community for postpartum women. After recruitment of the participants, an explanation and a consent of the study were obtained. In order to minimize selection bias, randomization was performed, and the subjects were then categorized into the wrist stabilization exercise + taping therapy (WSE + TT), wrist stabilization exercise (WSE), and control group, with 15 subjects in each group. All subjects visited G University on the first day (Monday) of the study participation to perform interventions in their assigned group. Subjects received individual training on intervention methods from the researchers and also performed exercises under the supervision of a physical therapist. Subjects performed the trained exercise intervention by themselves for another 4 days in their home. All participants visited G University every Monday for 8 weeks and were checked for individual pain and disability. The research team trained the optimal exercise method according to the individual condition of the subject, and the participant performed it the same for the remaining 4 days. Subjects received a daily phone call from the researcher to confirm whether or not they performed the exercise intervention and also asked questions about interventions or physical problems of the wrist. None of the subjects participating in this study were dropped out due to not performing more than two exercises. A total of 45 subjects received a measurement 1 day before the intervention and 1 day after the final intervention (Figure 1).

### 2.3. Intervention

The intervention method was determined by three physical therapists with more than 5 years clinical experience in musculoskeletal rehabilitation and two professors in the department of physical therapy based on clinical experience and previous research and reference.

The WSE + TT and WSE groups performed wrist stabilization exercises for 40 min a day for 8 weeks, using elastic bands and dumbbells. After fixing the elastic band to a ring, an isometric contraction exercise was performed for the wrist flexor and extensor and radial- and ulnar-deviation muscles in a sitting position so that the elastic band was at the front, back, left, and right sides of the body. In each direction, three sets of 10 repetitions were performed as a method of maintaining for 10 s against resistance, and a 1 min break between sets was provided. In addition, the dumbbell was held in the hand in a sitting position, and the arm was raised to the front, back, and sideways (shoulder flexion, extension, abduction, adduction) to the height possible while maintaining the wrist in a neutral position for 10 s. Each was performed 10 times. Thera-Band (TheraBand, Akron, OH, USA) offers a different amount of resistance depending on the band color. Therefore, the Thera-Band band of appropriate strength was selected for each patient by the therapist. Dumbbells were gradually increased from 0.5 kg to 2 kg according to the patient’s condition every week (Supplemental Figure S1). The control group performed passive range of motion (P-ROM) exercise for shoulder flexion, extension, abduction, and adduction, wrist flexion, extension, and radial and ulnar deviation instead of stabilization exercise for the same time as the other groups for the same time period (Supplemental Figure S2).

For taping, an elastic tape of about 60–80 cm in length was used depending on the length of the forearm, and with the muscle stretched as much as possible, the wrist extensor and flexor muscles were attached from the origin to the insertion site of the muscle. An elastic tape of approximately 15 cm in length was wound around the wrist one round starting from the radial side of the wrist (Supplemental Figure S3).

### 2.4. Outcome Measurements

Participants were evaluated before and one day after the final intervention by three well-trained physical therapists, who did not have any information on the subjects and the purpose of this study. The 100-mm VAS was used to measure the degree of pain, and it is a clinical tool that is widely used to measure subjective pain in the clinic and in researches [10,14,21]. The VAS subjectively displays the pain intensity that the patient feels by using a 100-mm horizontal line marked from 0 to 100 from left to right, with 0 meaning no pain and 100 meaning maximum pain.

The Disabilities of the Arm, Shoulder and Hand (DASH) questionnaire was used to assess wrist dysfunction. The DASH questionnaire is the most widely used outcome assessment tool in hand and arm surgeries worldwide and consists of 30 questions on a 5-point ranking scale that evaluate motion performance. The total score is 100 points, and the higher the score, the more severe the dysfunction. The internal consistency of the Korean version of the DASH was 0.94 [22].

In order to evaluate the quality of life, we used the Short Form 36 (SF-36) questionnaire, which is commonly used to evaluate the person’s quality of life in clinical practice [23]. The SF-36 is a 36-item health survey that consists of eight scales for physical and mental health. Physical health consists of physical functioning, role–physical, bodily pain, and general health, and mental health includes vitality, social functioning, role–emotional, and mental health. Each question consists of 2 to 6 points, and the higher the score, the higher the quality of life [24].

### 2.5. Data Analysis

SPSS 21.0 (IBM, Armonk, NY, USA) was used for all statistical analysis, and the normality test of variables was performed using the Shapiro–Wilk test. For the difference in variance among the three groups before and after the training, one-way ANOVA was used. For the post hoc analysis, the Bonferroni test was used. The paired *t*-test was used for within-group comparison. The statistical significance level of all data was less than 0.05.

## 3. Results

### 3.1. General Characteristics of Subjects

In this study, 45 postpartum women with wrist pain completed the pre- and post-test. The general characteristics are shown in Table 1. There was no significant difference in baseline between the groups.

### 3.2. Changes of Pain and Disability after Intervention

**Figure 2:**
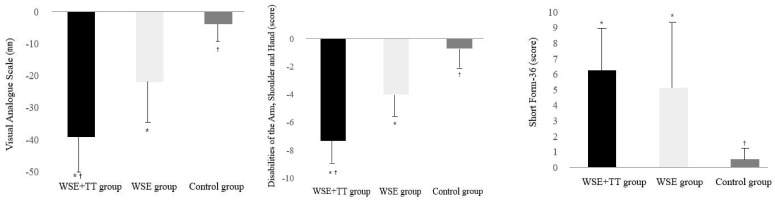
Changes of pain, disability, and quality of life following the intervention. * Significant difference compared to the control group (*p* < 0.05). ^†^ Significant difference compared to the WSE group (*p* < 0.05).

The pain and disability in the WSE + TT group (change values, −39.20 ± 11.21 mm, −7.33 ± 3.96 score, respectively) showed significant decreases compared to that of the WSE group (change values, −21.93 ± 15.35 mm, −4.00 ± 3.14 score, respectively) and the control group (change values, −3.87 ± 8.31 mm, −0.67 ± 2.09 score, respectively) after the intervention (Table 2, Figure 2).

### 3.3. Changes of Quality of Life after Intervention

The SF-36 score was significantly different among the three groups, and that of the WSE + TT group (change values, 6.27 ± 3.22 score) and the WSE group (change values, 5.13 ± 4.12 score) showed significantly greater improvement than that of the control group (change values, 0.53 ± 1.30 score) after the training. However, SF-36 scores were not significantly different between the WSE + TT and WSE groups after the intervention (Table 2, Figure 2).

## 4. Discussion

This study investigated the effects of wrist stabilization exercise combined with taping on wrist pain, functional disability, and quality of life in postpartum women with wrist pain. In our results, the WSE + TT group, which performed taping and wrist stabilization exercises, showed significant decreases in wrist pain and functional disability compared to the WSE and control groups, and a significant difference was also found between the WSE and control groups. Joint instability due to the increased ligament laxity causes traumatic damage to the joint tissues and musculoskeletal pain [25]. A previous study reported that patients with joint instability disorders have impaired proprioception, and exercise intervention could alleviate joint instability and hypermobility [26]. Daman et al. suggested that stabilization exercise reduced the degree of pain by improving proprioceptive sensation and joint stability [27].

Similar to these results, present studies also show the significant relief of pain in two groups performing stabilization exercises, and taping interventions made this effect more evident. In a study comparing the effect of taping and elastic bandage on wrist-joint-position sensation, it was found that the group who applied taping significantly improved the proprioception compared to the group who applied the elastic bandage [28]. External supports, such as taping and elastic bandages, improve joint movements by fixing or supporting unstable joints because they could affect the mechanoreceptors or stimulate proprioception in the application site [29]. Long et al. reported that the proprioceptive performance was significantly improved following taping application to the ankle joint in the weight-bearing condition, and they suggested that this is significantly correlated with an increase in the “comfort” felt by the participants [30]. They speculated that the central nervous system interpreted the optimal mechanical receptor and proprioceptor loads given by taping as comfort.

In the case of patients with chronic ankle instability, the taping application to the ankle joint reduced postural sway and the muscle activity of the lower limb to maintain posture during the single leg balance test. Through this, they suggested that taping could be considered an option to safely restore physical activity by improving joint stability [31]. In this study, functional movement was improved by wrist stabilization exercise, and taping intervention enhanced this effect similar to the pain measurement situation. According to various studies and our experience, decreased muscle stiffness in surrounding muscles to which taping was applied and increased functional movement are commonly caused [32]. This may be due to the relief of unnecessary muscle tension by the stability provided by taping and also induced through stimulation of the muscle spindle and the Golgi tendon organs. Taping could also improve the functional movement of the joint by inducing strengthening and recovery of the muscles using the reciprocal inhibitory effect [33]. Several studies have reported the direct and indirect effects of taping intervention on increasing muscle strength [34,35], which is suggested to be the result of spatial summation from a large number of presynaptic nerve fibers at the taping attachment site and stimulating the gamma motor nerve of the skeletal muscle under the attached skin of the taping to increase the tension of the fiber itself. In a study in which taping was applied to patients with carpometacarpal joint injuries, it was reported that there was an effect of reducing pain and enhancing muscle strength [36], and Cho et al. [37] reported that the grip strength and muscle activity improved as a result of taping in patients with lateral epicondylitis. However, according to a previous review study on the therapeutic effect of taping, only-taping intervention cannot show the effects of physical therapy modalities such as electrical stimulation or therapeutic exercise. It has been suggested that taping intervention is effective in reducing pain and improving movement when applied in combination with physical therapy modalities [15]. It has been reported that applying repetitive exercises in parallel with taping relieves muscle fatigue and muscle damage [38], and the results of this study are thought to be due to the combined effect of exercise rather than taping alone. Therefore, the effect of taping is believed to be more effective when combined with exercise than when applied alone.

This study also investigated the changes in quality of life after training. The SF-36 score of the WSE + TT group and WSE groups showed a significant improvement compared to that of the control group. Body dysfunction and disability are considered a risk factor for deteriorating subjective and mental health, and the risk of depression increases with functional limitations after childbirth [16]. Several studies reported that the quality of life also significantly improved as the pain decreased, and the function improved through stabilization exercises [39,40]. In this study, the significant improvement in the SF-36 scores of the WSE + TT and WSE groups compared to that in the control group is thought to be due to the reduction of negative emotions such as discomfort and stress through the reduction of physical symptoms. However, there was no significant difference in the SF-36 score between the WSE + TT group and the WSE group, which is thought to be due to the fact that the SF-36 was less sensitive to the measurement of the function of the upper limb, and the difference in the degree of improvement between groups was not large in the mental health part.

This study investigated the effects of wrist stabilization exercise combined with taping on wrist pain, functional disability, and quality of life in postpartum women with wrist pain. However, this study has some limitations. This study was designed by calculating the number of samples according to G-power analysis, but nonetheless, it is difficult to generalize the results of the study due to the small number of subjects. In addition, the level of activity, time, and occupation related to childcare or housework were not analyzed, so the influence of other factors that may affect wrist pain was not considered. In future studies, it is necessary to classify groups according to pain, dysfunction, or activity level of the wrist (whether breastfeeding, time to hold a baby, etc.) and compare the effects of taping combined with wrist stabilization exercise. In addition, a long-term follow-up study is needed to determine the duration of the effects of wrist stabilization exercise with/without taping intervention on pain and function in postpartum women.

## 5. Conclusions

This study demonstrated that wrist stabilization exercise combined with taping is effective in reducing the degree of pain and disability in postpartum women with wrist pain. Therefore, we suggest that wrist stabilization exercise and taping intervention can be effectively used in hospitals and facilities managing postpartum women to control wrist pain and functional impairment.

## Figures and Tables

**Table 1:** Common and clinical characteristics of the subjects (*n* = 45).

Variables	WSE + TT Group (*n* = 15)	WSE Group (*n* = 15)	Control Group (*n* = 15)	*p*
Age (years)	31.60 ± 4.64	31.47 ± 5.24	30.13 ± 6.02	0.709
Height (cm)	163.33 ± 5.34	161.80 ± 5.86	162.13 ± 5.07	0.720
Weight (kg)	57.47 ± 5.50	56.07 ± 4.17	54.67 ± 5.35	0.324
Duration of wrist pain (months)	5.93 ± 2.28	6.27 ± 2.84	6.00 ± 3.68	0.949

Wrist stabilization exercise + taping therapy (WSE + TT), wrist stabilization exercise (WSE). Values are expressed as the mean ± standard deviation (SD).

**Table 2:** Comparison pre-test and post-test among the three groups (*n* = 45).

	WSE + TT Group			WSE Group			Control Group			*p*
	Pre-Test	Post-Test	ES	Pre-Test	Post-Test	ES	Pre-Test	Post-Test	ES	
VAS (mm)	64.53 ± 9.26	25.33 ± 9.76 *	−4.11	59.60 ±13.86	37.67 ± 16.12 *	−1.45	60.00 ± 13.30	56.13 ± 11.83	−0.31	0.001
DASH (score)	41.40 ± 2.82	34.07 ± 3.77 *	−2.15	39.40 ±4.15	35.40 ± 3.94 *	−0.98	40.27 ± 3.90	39.60 ± 4.72	−0.15	<0.001
SF-36 (score)	69.47 ± 6.29	75.73 ± 4.71 *	−1.1	70.33 ± 8.23	75.47 ± 6.50 *	−0.68	68.27 ± 6.23	68.80 ± 6.10	−0.09	<0.001

Values are expressed as the mean ± standard deviation (SD). * Significant differences between pre- and post-test (*p* < 0.05). Effect size (ES); visual analogue scale (VAS); Disabilities of the Arm, Shoulder and Hand (DASH); Short Form-36 (SH-36). Wrist stabilization exercise + taping therapy (WSE + TT), wrist stabilization exercise (WSE).

## Data Availability

Data will be available on reasonable request by email to Tae-Sung In (20160072@gimcheon.ac.kr).

## Supplementary Materials

The following are available online at https://www.mdpi.com/article/10.3390/ijerph18073564/s1, Figure S1: Wrist stabilization exercise in groups WSE + TT and WSE. Figure S2: P-ROM exercise in control group. Figure S3: Taping application in WSE + TT group.

## References

[1] Weis C.A., Pohlman K., Draper C., da Silva-Oolup S., Stuber K., Hawk C. (2020). Chiropractic Care of Adults with Postpartum-Related Low Back, Pelvic Girdle, or Combination Pain: A Systematic Review. J. Manip. Physiol. Ther..

[2] Kisner C., Colby L.A. (2012). Therapeutic Exercise: Foundations and Techniques.

[3] Webb B.G., Retting L.A. (2008). Gymnastic wrist injuries. Curr. Sports Med. Rep..

[4] Sit R.W.S., Tam W.H., Chan D.C.C. (2017). A Pilot Cross-Sectional Study of Postpartum Wrist Pain in an Urban Chinese Population: Its Prevalence and Risk Factors. Pain Physician.

[5] Cherni Y., Desseauve D., Decatoire A., Veit-Rubinc N., Begon M., Pierre F. (2019). Evaluation of ligament laxity during pregnancy. J. Gynecol. Obstet. Hum. Reprod..

[6] Long G., Yaoyao Z., Na Y., Ping Y., Mingsheng T. (2021). Generalized joint laxity as a predictor of recovering from low back pain during pregnancy—A prospective study. J. Orthop. Sci..

[7] Skoff H. (2001). “Postpartum/newborn” de Quervain’s tenosynovitis of the wrist. Am. J. Orthop. (Belle Mead NJ).

[8] Prosser R., Hancock M.J., Nicholson L.L., Harvey L.A., LaStayo P., Hargreaves I., Herbert R. (2012). Prognosis and prognostic factors for patients with persistent wrist pain who proceed to wrist arthroscopy. J. Hand Ther..

[9] Leanderson J., Ekstam S., Salomonsson C. (1996). Taping of the ankle-the effect on postural sway during perturbation, before and after a training session. Knee Surg. Sports Traumatol. Arthrosc..

[10] Lee H., Lim H. (2020). Effects of Double-Taped Kinesio Taping on Pain and Functional Performance due to Muscle Fatigue in Young Males: A Randomized Controlled Trial. Int. J. Environ. Res. Public Health.

[11] Lee D., Bae Y. (2021). Short-Term Effect of Kinesio Taping of Lower-Leg Proprioceptive Neuromuscular Facilitation Pattern on Gait Parameter and Dynamic Balance in Chronic Stroke with Foot Drop. Healthcare.

[12] Choi S.H., Lim C.G. (2020). Immediate Effects of Ankle Non-elastic Taping on Balance and Gait Ability in Patients With Chronic Stroke: A Randomized, Controlled Trial. J. Manip. Physiol. Ther..

[13] Giray E., Karali-Bingul D., Akyuz G. (2019). The Effectiveness of Kinesiotaping, Sham Taping or Exercises Only in Lateral Epicondylitis Treatment: A Randomized Controlled Study. PM&R.

[14] Kim G.S., Weon J.H., Kim M.H., Koh E.K., Jung D.Y. (2020). Effect of weight-bearing wrist movement with carpal-stabilizing taping on pain and range of motion in subjects with dorsal wrist pain: A randomized controlled trial. J. Hand Ther..

[15] Farhadian M., Morovati Z., Shamsoddini A. (2019). Effect of Kinesio Taping on Pain, Range of Motion, Hand Strength, and Functional Abilities in Patients with Hand Osteoarthritis: A Pilot Randomized Clinical Trial. Arch. Bone Jt. Surg..

[16] Menta R., Randhawa K., Côté P., Wong J.J., Yu H., Sutton D., Varatharajan S., Southerst D., D’Angelo K., Cox J. (2015). The Effectiveness of Exercise for the Management of Musculoskeletal Disorders and Injuries of the Elbow, Forearm, Wrist, and Hand: A Systematic Review by the Ontario Protocol for Traffic Injury Management (OPTIMa) Collaboration. J. Manip. Physiol. Ther..

[17] Richardson C., Hodges P., Hides J. (2004). Therapeutic Exercise for Lumbopelvic Stabilization: A Motor Control Approach for the Treatment and Prevention of Low Back Pain.

[18] Shin H.J., Jung J.H., Kim S.H., Hahm S.C., Cho H.Y. (2020). A Comparison of the Transient Effect of Complex and Core Stability Exercises on Static Balance Ability and Muscle Activation during Static Standing in Healthy Male Adults. Healthcare.

[19] Gomes-Neto M., Lopes J.M., Conceição C.S., Araujo A., Brasileiro A., Sousa C., Carvalho V.O., Arcanjo F.L. (2017). Stabilization exercise compared to general exercises or manual therapy for the management of low back pain: A systematic review and meta-analysis. Phys. Ther. Sport..

[20] Alghadir A.H., Anwer S., Iqbal A., Iqbal Z.A. (2018). Test–retest reliability, validity, and minimum detectable change of visual analog, numerical rating, and verbal rating scales for measurement of osteoarthritic knee pain. J. Pain Res..

[21] Alotaibi A., Petrofsky J., Daher N.S., Lohman E., Syed H.M., Lee H. (2020). The Effect of Monophasic Pulsed Current with Stretching Exercise on the Heel Pain and Plantar Fascia Thickness in Plantar Fasciitis: A Randomized Controlled Trial. Healthcare.

[22] Lee J.Y., Lim J.Y., Oh J.H., Ko Y.M. (2008). Cross-cultural adaptation and clinical evaluation of a Korean version of the disabilities of arm, shoulder, and hand outcome questionnaire (K-DASH). J. Shoulder Elbow Surg..

[23] Lee D., Kim S.J., Kim H. (2020). A 12 week, randomized, double-blind, placebo-controlled clinical trial for the evaluation of the efficacy and safety of HT083 on mild osteoarthritis. Medicine.

[24] Brazier J.E., Harper R., Jones N.M., O’Cathain A., Thomas K.J., Usherwood T., Westlake L. (1992). Validating the SF-36 health survey questionnaire: New outcome measure for primary care. BMJ.

[25] Sahin N., Baskent A., Ugurlu H., Berker E. (2008). Isokinetic evaluation of knee extensor/flexor muscle strength in patients with hypermobility syndrome. Rheumatol. Int..

[26] Ferrell W.R., Tennant N., Sturrock R.D., Ashton L., Creed G., Brydson G. (2004). Amelioration of symptoms by enhancement of proprioception in patients with joint hypermobility syndrome. Arthritis Rheum..

[27] Daman M., Shiravani F., Hemmati L., Taghizadeh S. (2019). The Effect of Combined Exercise Therapy on Knee Proprioception, Pain Intensity and Quality of Life in Patients With Hypermobility Syndrome: A Randomized Clinical Trial. J. Bodyw. Mov. Ther..

[28] Ucuzoglu M.E., Unver B., Sarac D.C., Cilga G. (2020). Similar effects of two different external supports on wrist joint position sense in healthy subjects: A randomized clinical trial. Hand Surg. Rehabil..

[29] Miralles I., Monterde S., Montull S., Salvat I., Fernández-Ballart J., Beceiro J. (2010). Ankle taping can improve proprioception in healthy volunteers. Foot Ankle Int..

[30] Long Z., Wang R., Han J., Waddington G., Adams R., Anson J. (2017). Optimizing ankle performance when taped: Effects of kinesiology and athletic taping on proprioception in full weight-bearing stance. J. Sci. Med. Sport.

[31] Sarvestan J., Ataabadi P.A., Svoboda Z., Kovačikova Z., Needle A.R. (2020). The effect of ankle Kinesio taping on ankle joint biomechanics during unilateral balance status among collegiate athletes with chronic ankle sprain. Phys. Ther. Sport.

[32] Chao Y.W., Lin J.J., Yang J.L., Wang W.T. (2016). Kinesio taping and manual pressure release: Short-term effects in subjects with myofasical trigger point. J. Hand Ther..

[33] Perrin D.H. (2005). Athletic Taping and Bracing.

[34] Halseth T., McChesney J.W., DeBeliso M., Vaughn R., Lien J. (2004). The effects of kinesio taping on proprioception of the ankle. J. Sports Sci. Med..

[35] Kase K., Wallis J., Kase T. (2003). Clinical Therapeutic Applications of the Kinesio Taping Method.

[36] Braakman M., Oderwal E.E., Haentjens M.H. (1998). Functional taping of fractures of the 5th metacarpal results in a quicker recovery. Injury.

[37] Shelton G.L. (1992). Conservative management of patellofemoral dysfunction. Prim. Care.

[38] Cho Y.T., Hsu W.Y., Lin L.F., Lin Y.N. (2018). Kinesio taping reduces elbow pain during resisted wrist extension in patients with chronic lateral epicondylitis: A randomized, double-blinded, cross-over study. BMC Musculoskelet. Disord..

[39] Zhang S., Fu W., Pan J., Wang L., Xia R., Liu Y. (2016). Acute effects of Kinesio taping on muscle strength and fatigue in the forearm of tennis players. J. Sci. Med. Sport.

[40] Woolhouse H., Gartland D., Perlen S., Donath S., Brown S.J. (2014). Physical health afterchildbirth and maternal depression in the first 12 months post partum: Results of an Australian nulliparous pregnancy cohort study. Midwifery.

