# Supplementary material for: The Effects of Taping Combined with Wrist Stabilization Exercise on Pain, Disability, and Quality of Life in Postpartum Women with Wrist Pain: A Randomized Controlled Pilot Study
Source: Int J Environ Res Public Health. 2021 Mar 30;18(7):3564. doi: 10.3390/ijerph18073564 (PMC8037921; doi:10.3390/ijerph18073564)
Supplement: Supplementary file 1 [file ijerph-18-03564-s001.pdf]

Supplemental Figure S1. Wrist stabilization exercise in groups WSE+TT and WSE

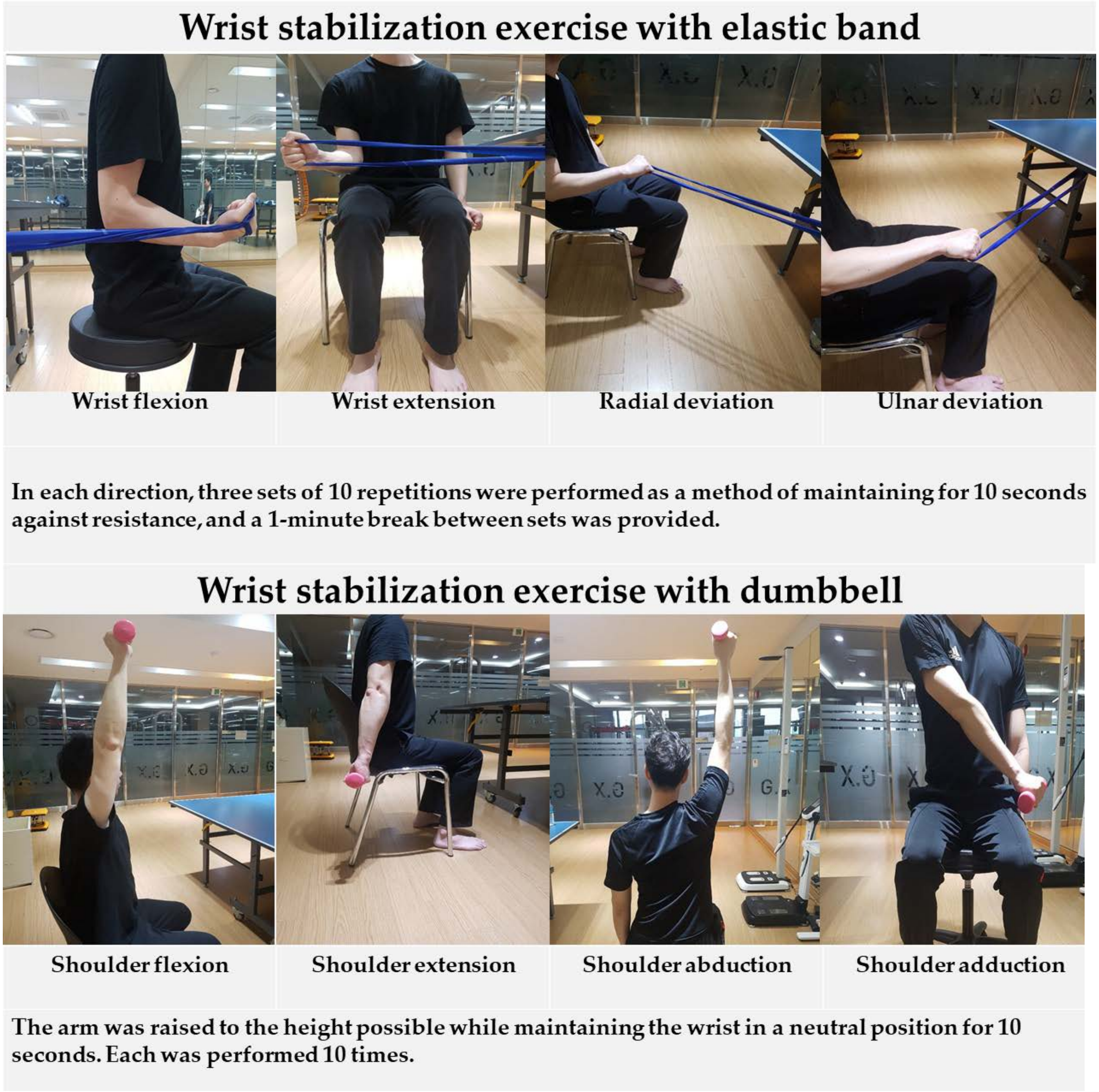

Supplemental Figure S2. P-ROM exercise in Control group

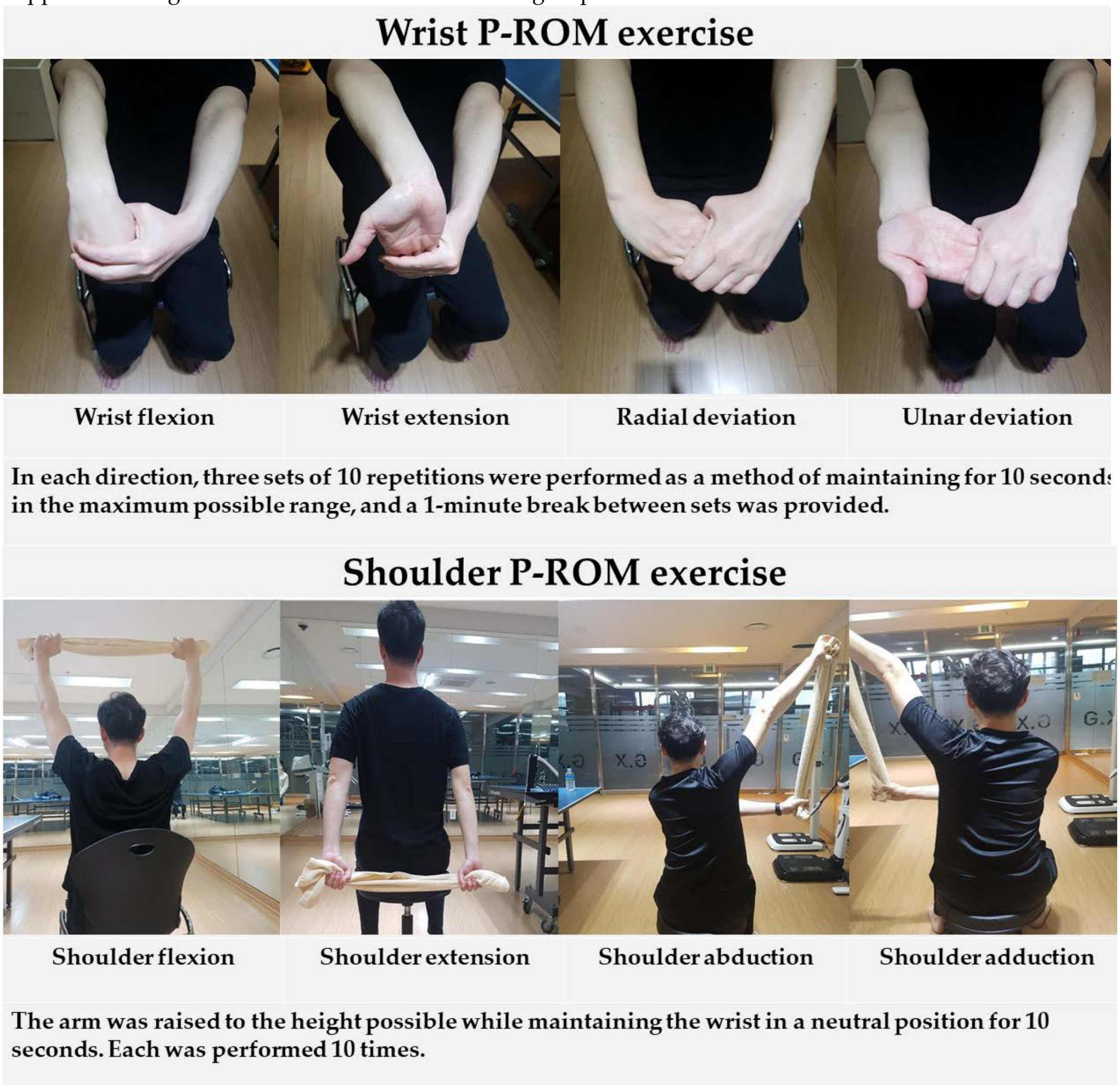

Supplemental Figure S3. Taping application in WSE+TT group

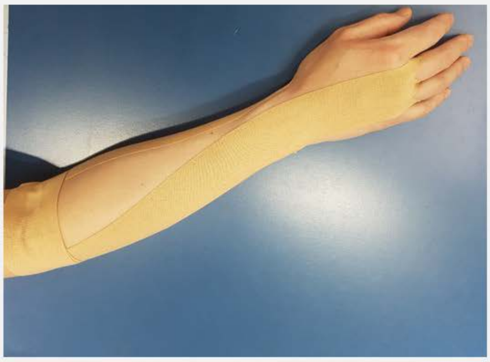

Kinesio tape was applied to the origin and insertion site of the muscle with the flexors and extensors of the wrist stretched as far as possible. In addition, 15cm kinesio tape was wound around the wrist from the radial side to the ulna side and attached.
